# Percent Tolerance to Phosphorus Deficiency (PTPD) as a Potential Metric for Genotypic Screening in Soybean (*Glycine max* L.)

**DOI:** 10.3390/plants15152408

**Published:** 2026-08-06

**Authors:** Jing Zhao, Debin Yu, Demin Rao, Hongtao Wang, Ziru Hao, Qiang Qiu, Yinkai Zhao, Xiaohui Wang, Tong Cheng, Xiujuan Yan, Minghao Zhang, Botao Cong, Mingshu Li, Fangang Meng, Wei Zhang

**Affiliations:** 1 Soybean Research Institute, Jilin Academy of Agricultural Sciences, Changchun 130033, China; zhaojing@jaas.com.cn (J.Z.); yudebin@jaas.com.cn (D.Y.); raodemin@jaas.com.cn (D.R.); chengtong@jaas.com.cn (T.C.); zhangminghao@jaas.com.cn (M.Z.);; 2 Jilin Provincial Service Center for Agricultural and Rural Talent Development, No. 6152, Ziyou Road, Nanguan District, Changchun 130022, China; 17549639186@163.com (H.W.);

**Keywords:** *Glycine max* L., phosphorus deficiency, percent tolerance to phosphorus deficiency (PTPD), principal component analysis, comprehensive evaluation, OJIP fluorescence transient, grain phosphorus utilization efficiency

## Abstract

Phosphorus (P) deficiency severely limits soybean (*Glycine max* L.) productivity. This study proposed a three-stage screening framework to identify reliable traits and P-efficient genotypes. In Experiment I, percent tolerance to phosphorus deficiency (PTPD) was calculated for ten growth parameters across 98 genotypes under P-deficient and control conditions. Principal component analysis and comprehensive evaluation identified six key indicators in Experiment I, which were subsequently refined to five indicators through further analysis: SPAD at V3 and R1, photosynthetic rate at R1, shoot dry weight at R8, and seed number per plant at R8. Experiment II re-evaluated these traits using 12 contrasting genotypes under three P levels, identifying CN 15 as the most P-efficient and SN 22 as the most P-inefficient. Experiment III further revealed that CN 15 maintained superior PSII performance and exhibited a 26.2% increase in grain P-utilization efficiency under 0 µM KH_2_PO_4_ treatment. This integrated framework offers a preliminary reference for screening P-efficient soybean genotypes under controlled conditions, pending field evaluation.

## 1. Introduction

Phosphorus (P) is an indispensable macronutrient critical for plant growth and development [1], participating in a wide array of cellular processes including metabolic regulation, photosynthesis, respiration, and the biosynthesis of membrane phospholipids [2,3]. Soybeans (*Glycine max* L.) exhibit a high demand for phosphorus to achieve optimal seed yield and quality [4]. Phosphorus deficiency frequently manifests during early developmental stages rather than at maturity [4,5], leading many researchers to focus on the seedling phase as the key period for assessing phosphorus tolerance [6,7]. In soybean, phosphorus deficiency during early growth can significantly reduce seedling biomass, phosphorus concentration [8], leaf area [9], the number of nodes and seeds [10], and these detrimental effects may persist even after phosphorus availability is restored [11].

The primary source of phosphorus for plants is inorganic phosphate (Pi) [12], mainly in the form of H_2_PO_4_^−^ at neutral pH, which is actively transported into plant cells [2]. Despite its low concentration in soil solution (typically 1–10 µM), the internal Pi concentration in plant tissues is considerably higher, approximately 5–20 mM [13]. In alkaline soils, Pi tends to form complexes with calcium, whereas in acidic soils, it is often bound to aluminum or iron [13,14]. Furthermore, soil organic components, such as fertilizers and crop residues, particularly phytic acids (inositol compounds), can also sequester phosphates [15].

Phosphorus efficiency (PE) is a complex trait that can be dissected into two major components: phosphorus uptake efficiency (PUpE, the capacity to acquire inorganic phosphate from the soil) and internal phosphorus utilization efficiency (PUtE, the ability to produce biomass or yield per unit of tissue phosphorus) [14]. Phosphorus deficiency tolerance is a broader agronomic concept that encompasses the overall ability of a genotype to maintain growth and yield under low-P stress; it can be achieved through superior PUpE, superior PUtE, or a combination of both. To cope with phosphorus deficiency, plants have evolved an array of physiological and morphological adaptations that enhance phosphorus acquisition (i.e., phosphorus uptake efficiency, PUpE) and improve internal utilization efficiency (PUtE) [16,17,18]. These strategies, which primarily enhance PUpE, include forming symbiotic associations, modifying root architecture, and exuding organic acids and acid phosphatases from roots [19]. In this context, the cultivation of soybean varieties with high PE—defined here as the ability to sustain high biomass or grain yield under limited phosphorus availability—holds significant potential for enhancing soybean production [20,21]. Genotypic variation in both PUpE and PUtE has been documented across multiple crops [7], including soybean [4,21,22,23], rice (*Oryza sativa* L.) [24,25,26], Brassica (*Brassica napus* L.) [27], and wheat (*Triticum aestivum* L.) [28]. Vandamme et al. [21] observed that phosphorus uptake (a measure of PUpE) varied little among soybean genotypes under high phosphorus availability but showed a two-fold difference under low phosphorus conditions. While enhanced PUpE is recognized as a key adaptive trait, particularly in soils with high phosphorus retention capacity [29,30], superior PUtE may also contribute to reduced soil phosphorus extraction, especially in low-input agricultural systems [31,32]. Early root growth and the relative amount of seed-borne phosphorus reserves are potential determinants of phosphorus deficiency tolerance(i.e., the ability to maintain growth and yield under low P stress) [33,34].

Various stress indices have been proposed to quantify crop responses to environmental constraints, such as the stress susceptibility index (SSI) and the stress tolerance index (STI) [35]. These indices typically incorporate the population mean into their calculation and are thus sensitive to the composition of the evaluated panel. In the present study, we adopted the percent tolerance to phosphorus deficiency (PTPD) as the primary screening metric. PTPD expresses the response of each genotype solely as a proportion of its own control performance, making it more intuitive and independent of the group of accessions under evaluation. Although direct comparisons with STI or SSI were not a primary objective, this approach emphasizes relative stability and avoids the confounding effect of population-dependent ranking. Since individual growth parameters often respond inconsistently to low-P stress across genotypes, reliance on a single trait may lead to misclassification. This underscores the need for an integrated metric. We hypothesized that PTPD, derived from multi-trait PCA, could effectively rank soybean genotypes for P efficiency, and that shoot-based traits would serve as reliable proxies under P deficiency.

This study aims to: (1) screen for soybean genotypes exhibiting high phosphorus efficiency, and (2) identify key PE-related traits that can facilitate the development of high-efficiency soybean varieties through breeding programs.

## 2. Results

### 2.1. Experiment I: Preliminary Screening for Low-Phosphorus Tolerance

#### 2.1.1. Phenotypic Variation Under Phosphorus Deficiency

**Figure 1:**
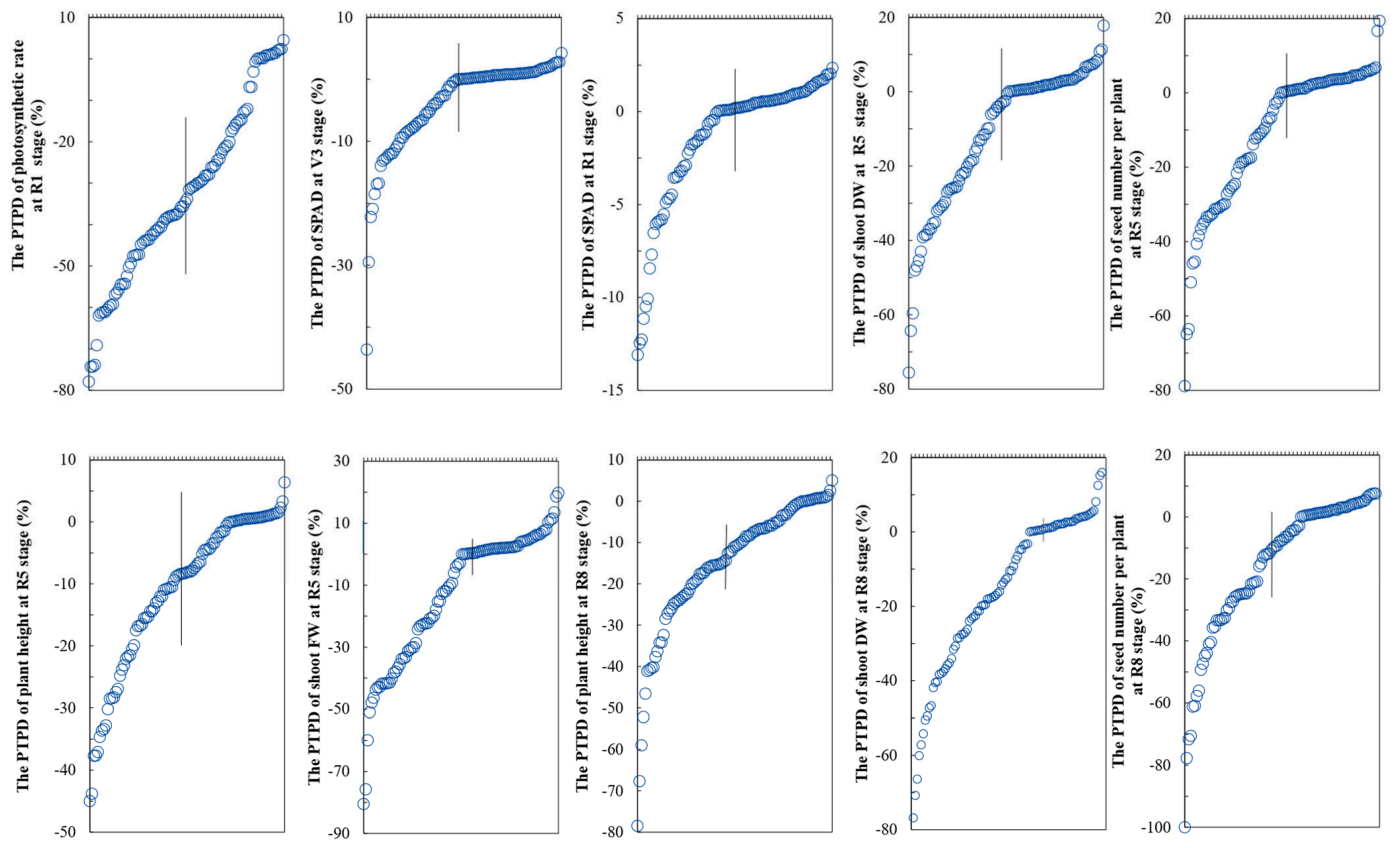
PTPD of ten growth parameters under P1 (0 µM KH_2_PO_4_). Each open circle represents one genotype. The LSD bar (α = 0.01) was derived from a one-way ANOVA model containing genotype as the sole fixed factor, performed separately on the PTPD values of each parameter, followed by Fisher’s LSD test for pairwise genotype comparisons within that parameter. This is a descriptive visualization of genotypic variation and does not include random effects. n = 3 biological replicates per genotype–treatment.

**Figure 2:**
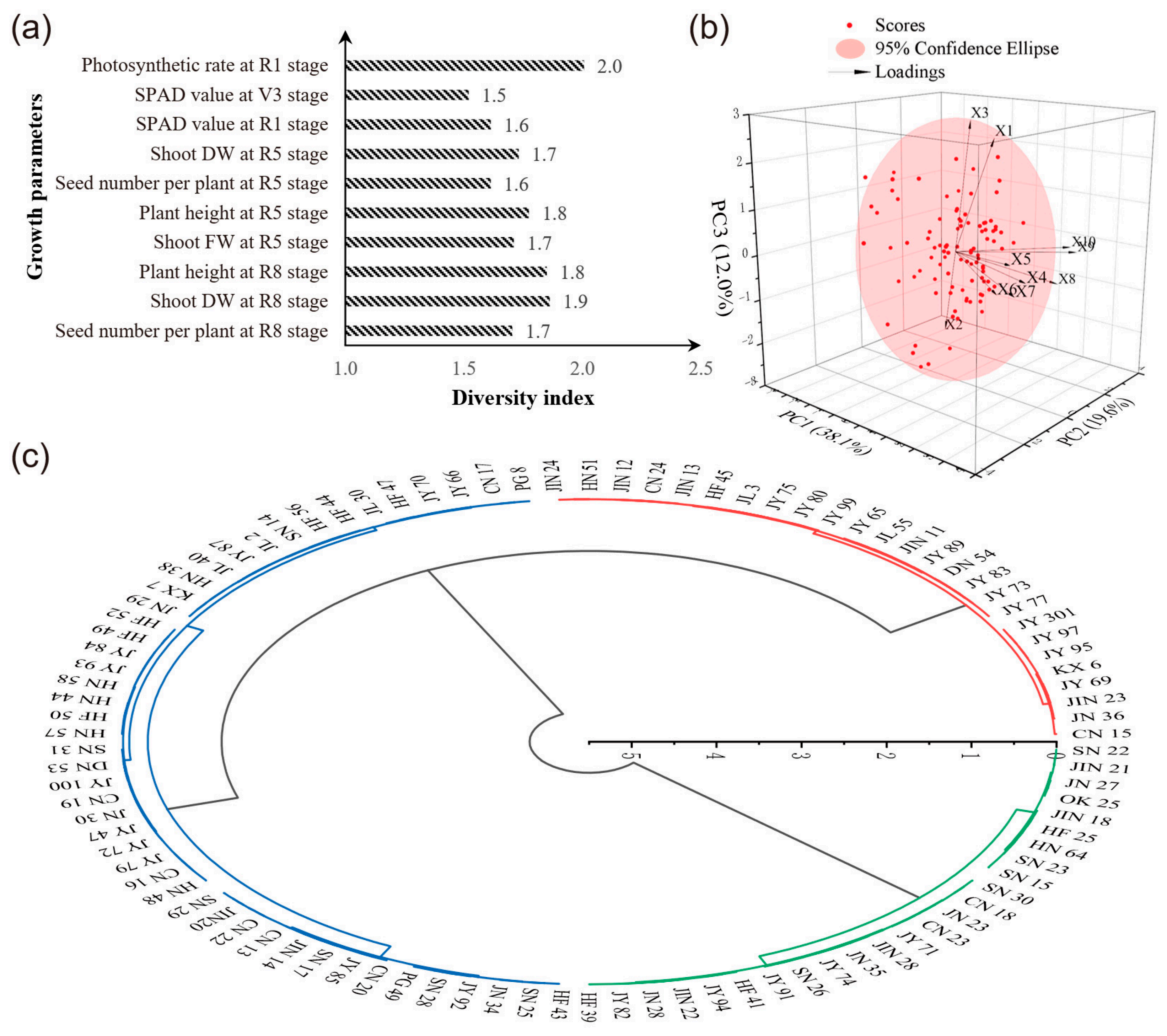
Phenotypic variation, principal component analysis, and genotypic clustering based on percent tolerance to phosphorus deficiency (PTPD) in 98 soybean (*Glycine max* L.) genotypes (Experiment I). (**a**) Diversity indices of the ten growth parameters, calculated as H′ = *–∑ p_k_ ln(p_k_)* using ten equal-interval classes across the PTPD range. Higher H′ indicates greater phenotypic variation. (**b**) Principal component analysis (PCA) of PTPD values. The parameters are: X1, photosynthetic rate at R1 stage; X2, SPAD value at V3 stage; X3, SPAD value at R1 stage; X4, shoot DW at R5 stage; X5, seed number per plant at R5 stage; X6, plant height at R5 stage; X7, shoot FW at R5 stage; X8, plant height at R8 stage; X9, shoot DW at R8 stage; X10, seed number per plant at R8 stage. (**c**) Hierarchical cluster dendrogram of comprehensive evaluation (CE) values using Ward’s method with squared Euclidean distance. The dendrogram was cut into three clusters based on phosphorus deficiency tolerance: strong (26, red), moderate (47, blue), and weak (25, green). Genotype identities are provided on the branches.

The effect of phosphorus deficiency stress on ten growth parameters across 98 soybean genotypes was evaluated by PTPD. As shown in Figure 1, PTPD values varied widely among genotypes for each parameter. Phosphorus deficiency (P1) significantly impaired multiple growth traits, with photosynthetic rate (X_1_) exhibiting the greatest average reduction of 32.4% relative to the control (Table 1). This parameter also displayed the highest diversity index (2.0) among all traits (Figure 2a), indicating extensive genotypic variation. SPAD values, shoot dry weight (DW), shoot fresh weight (FW), and seed number per plant were also markedly affected (Table 1). However, a small subset of genotypes showed positive PTPD values for certain traits, revealing genotypic differences in stress response and suggesting that no single parameter can reliably assess phosphorus deficiency tolerance.

#### 2.1.2. Principal Component Analysis and Identification of Key Traits

Principal component analysis (PCA) of PTPD values (Table 2) extracted five principal components (PCs) with eigenvalues > 1, which together explained 87.87% of the total variance (Figure 2b). The Kaiser–Meyer–Olkin (KMO) measure of sampling adequacy was 0.72, and Bartlett’s test of sphericity was significant (χ^2^ = 2317.4, *p* < 0.001), validating the suitability of the data for PCA.

The first principal component (C_1_) was dominated by shoot DW at R5 (X_4_) with the highest coefficient (0.473). The second component (C_2_) was primarily associated with shoot DW at R8 (X_9_) and seed number per plant at R8 (X_10_), with coefficients of 0.545 and 0.533, respectively. The third component (C_3_) was most strongly influenced by SPAD value at R1 (X_3_, coefficient 0.646) and photosynthetic rate at R1 (X_1_, 0.583). The fourth (C_4_) was loaded heavily by SPAD value at V3 (X_2_, 0.865), while the fifth (C_5_) showed relatively high coefficients for X_1_ (–0.663) and X_3_ (0.698). Based on the consistency of dominant loadings across the first four PCs, the following traits were identified as effective indicators for evaluating low-phosphorus tolerance: SPAD value at V3, photosynthetic rate and SPAD value at R1, shoot DW at R5, shoot DW at R8, and seed number per plant at R8.

#### 2.1.3. Membership Function Analysis and Comprehensive Evaluation

Membership function values (*M_ij_*) for each PC varied substantially among genotypes. For example, JN 36 achieved the maximum M_1_ value (1.000) in C1, indicating strong tolerance for traits associated with that component, whereas SN 22 scored the minimum (0), reflecting weak tolerance (Supplementary Table S1). Weight coefficients (*W_j_*) calculated from the variance contribution rates of the five PCs (38.1%, 19.6%, 12.0%, 9.9%, and 8.2%) were 0.434, 0.223, 0.137, 0.112, and 0.094, respectively (Table 2). The comprehensive evaluation (CE) values ranged from 0.393 (SN 22) to 0.856 (CN 15), enabling clear ranking of the genotypes (Supplementary Table S1).

#### 2.1.4. Cluster Analysis for Experiment I

Hierarchical cluster analysis was performed on CE values (Figure 2c). The optimal number of clusters was determined by examining the fusion coefficient, RSQ, and pseudo-F statistic (PSF). As shown in Supplementary Table S2, a sharp increase in the fusion coefficient (from 0.90 to 2.32) and a marked decline in RSQ (from 0.870 to 0.699) occurred when moving from three to two clusters, while the PSF remained high for the three-cluster solution (318.3). Based on these criteria, the dendrogram was cut to yield three groups: 26 genotypes were classified as having strong phosphorus deficiency tolerance, 47 as moderate, and 25 as weak.

### 2.2. Experiment II: Further Selection of Phosphorus-Efficient Genotypes

#### 2.2.1. Re-Evaluation of Screening Traits

The six most tolerant and six most sensitive genotypes from Experiment I were re-evaluated under three phosphorus levels (CK, P1, P2) using the six traits previously identified. PCA of the PTPD values for these six parameters (Table 3) generated four PCs with eigenvalues > 1, explaining 86.21% of the total variance. The first component (CC_1_) was dominated by shoot DW at R8 (Y_5_) and seed number per plant at R8 (Y_6_), with loadings above 0.59. The second (CC_2_) showed high loadings for photosynthetic rate at R1 (Y_1_, 0.600), SPAD at R1 (Y_3_, 0.470), and shoot DW at R5 (Y_4_, 0.514). The third (CC_3_) was mainly influenced by SPAD at V3 (Y_2_, 0.845), and the fourth (CC_4_) contrasted SPAD at R1 (Y_3_, –0.701) with other traits. Across the four components, five parameters—Y_1_, Y_2_, Y_3_, Y_5_, and Y_6_—consistently emerged as key contributors, each exhibiting a loading with an absolute value ≥ 0.6 on at least one of the first four principal components, so shoot dry weight at R5 (Y_4_) was not retained in the final indicator set. Moreover, retaining Y_4_ alongside Y_5_ would have over-represented biomass-accumulation traits in the final indicator set, as shoot DW was already captured by the agronomically more integrative variable Y_5_ at maturity.

#### 2.2.2. Comprehensive Evaluation and Classification

**Figure 3:**
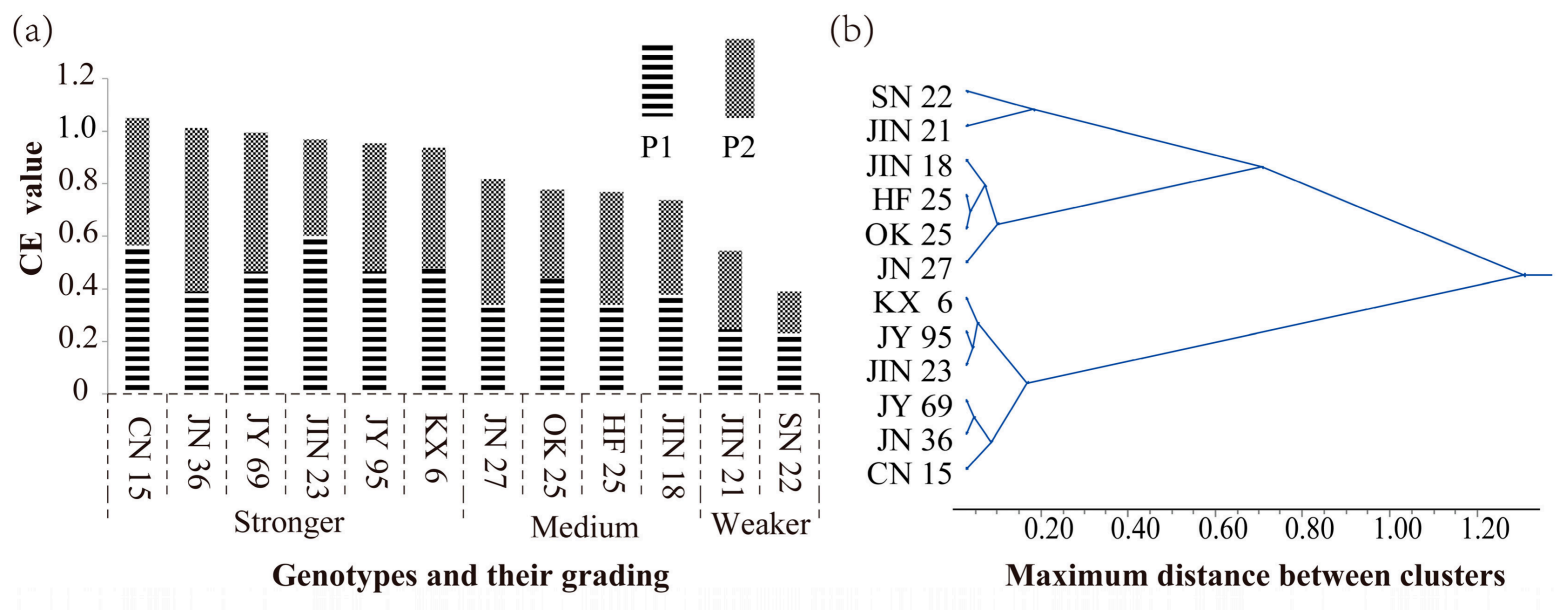
Comprehensive evaluation and cluster analysis of 12 selected soybean (*Glycine max* L.) genotypes under combined low-phosphorus treatments (Experiment II). (**a**) Comprehensive evaluation (CE) values under no-P (P1, 0 µM KH_2_PO_4_) and low-P (P2, 100 µM KH_2_PO_4_). Bars represent mean ± SD (n = 3 biological replicates). Stronger, medium, and weaker represent the genotypes’ tolerance grading to phosphorus deficiency according to CE in Experiment I. The overall ranking was based on the summed CE value of P1 + P2 (Table 4). (**b**) Hierarchical cluster dendrogram of summed CE values. Genotypes were classified as phosphorus-efficient (6), intermediate (4), or phosphorus-inefficient (2).

The comprehensive evaluation (CE) values were calculated separately for P1 and P2 treatments, and their sum was used to rank overall phosphorus efficiency. As shown in Table 4 and Figure 3a, SN 22 exhibited the lowest summed CE value (0.39), while CN 15 displayed the highest (1.06). Hierarchical clustering of the summed CE values separated the 12 genotypes into three groups (Figure 3b): SN 22 and JIN 21 were classified as phosphorus-inefficient; KX 6, JY 95, JIN 23, JY 69, JN 36, and CN 15 were classified as phosphorus-efficient; the remaining four genotypes showed intermediate tolerance. The optimal number of clusters was determined to be three based on the following criteria (Supplementary Table S3): (i) the fusion coefficient increased sharply from 0.154 to 0.677 (a 3.4-fold jump) when merging from three to two clusters, indicating that two highly dissimilar clusters would be forcibly combined; (ii) the RSQ dropped substantially from 0.947 to 0.664 at the same step, representing a loss of approximately 30% of the explained variance; and (iii) the pseudo-F statistic collapsed from 80.5 to 19.7.

### 2.3. Experiment III: Physiological Evaluation of Phosphorus Efficiency

#### 2.3.1. PSII Functionality Assessed by OJIP Fluorescence Transients

**Figure 4:**
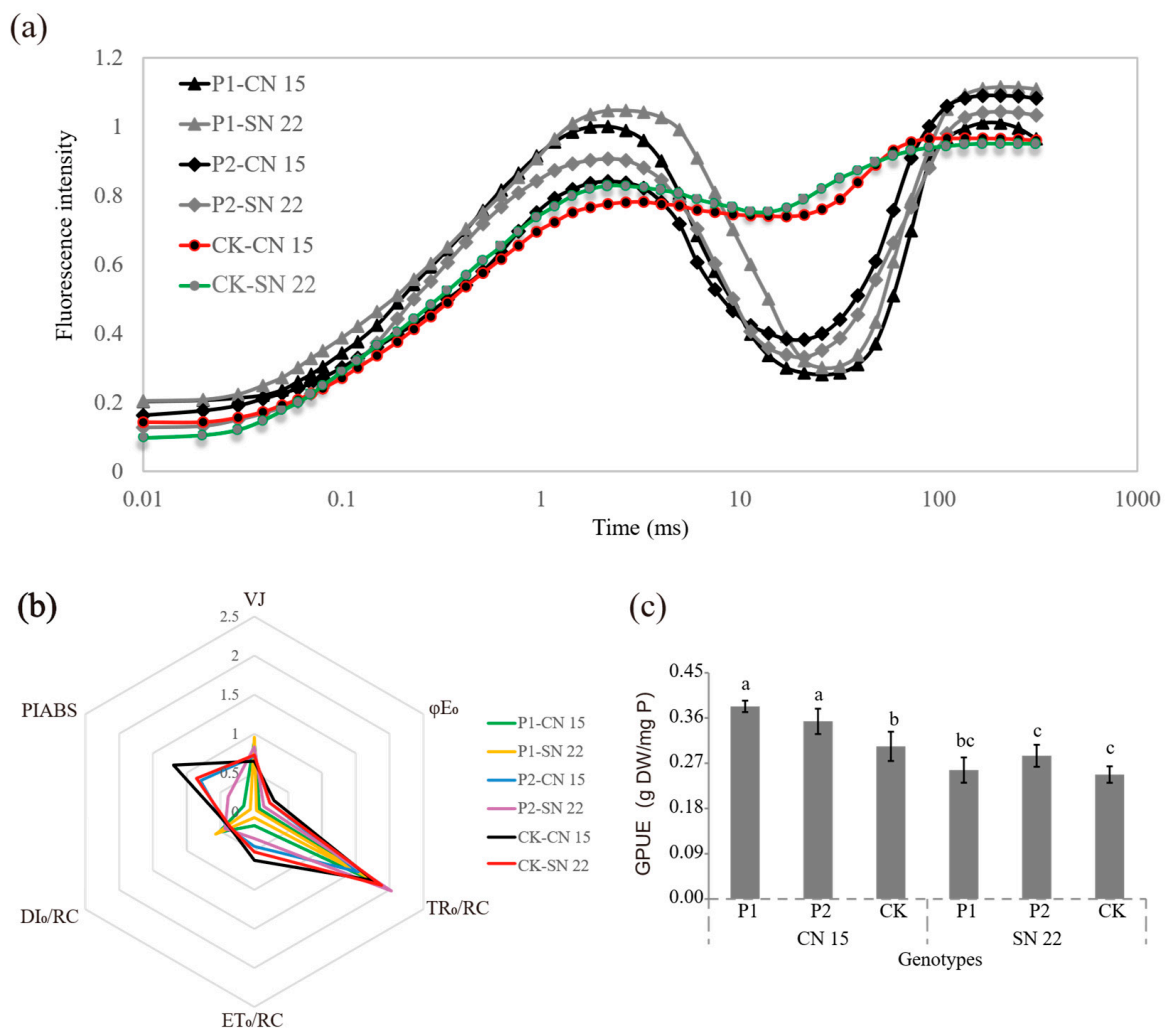
Physiological evaluation of phosphorus efficiency in contrasting soybean (*Glycine max* L.) genotypes (CN 15 vs. SN 22) under three P regimes (Experiment III). (**a**) OJIP fluorescence transients normalized as Vt = (Ft − F_0_)/(Fm − F_0_) and plotted on a logarithmic time scale. CK, 500 µM KH_2_PO_4_; P1, 0 µM; P2, 100 µM. (**b**) JIP-test parameters: TR_0_/RC (trapped energy flux per RC), ET_0_/RC (electron transport flux per RC), DI_0_/RC (dissipated energy flux per RC), φE_0_ (quantum yield of electron transport), VJ (relative variable fluorescence at J-step), and PIABS (performance index on absorption basis). (**c**) Grain phosphorus utilization efficiency (GPUE), calculated as grain dry weight (g plant^−1^) divided by grain P content (mg plant^−1^). Bars are means ± SD (n = 3). Split-plot ANOVA (genotype main plot, P subplot) revealed a significant interaction (*p* = 0.008). Different letters indicate significant differences among treatments within each genotype (Tukey’s HSD, *p* < 0.05).

To validate the screening results, CN 15 (highest CE) and SN 22 (lowest CE) were selected for detailed physiological analysis. Under adequate phosphorus supply (CK), both genotypes exhibited a typical O-J-I-P polyphasic rise in chlorophyll a fluorescence transient (Figure 4a). Phosphorus deficiency (P1, 0 µM Pi) induced a marked increase in relative variable fluorescence at the J-step (VJ) and the I-step, characteristic of impaired electron transport on the acceptor side of PSII. The intermediate P2 treatment (100 µM Pi) produced transient curves resembling those of CK in the phosphorus-efficient genotype (CN 15) but still deviated in the phosphorus-inefficient genotype (SN 22). Quantitative JIP-test analysis (Figure 4b) confirmed that CN 15 maintained significantly higher electron transport flux per reaction center (ET_0_/RC), quantum yield of electron transport (φE_0_), and performance index on an absorption basis (PIABS) than SN 22 under all phosphorus levels. These findings indicate superior PSII performance and more efficient conversion of absorbed light energy to photochemistry in the phosphorus-efficient genotype.

#### 2.3.2. Grain Phosphorus Utilization Efficiency

Grain phosphorus utilization efficiency (GPUE) was significantly influenced by genotype and its interaction with phosphorus treatment (split-plot ANOVA, interaction *p* = 0.008). As shown in Figure 4c, GPUE of SN 22 remained unchanged across P1 and P2 treatments compared with CK. In contrast, CN 15 exhibited a significant increase in GPUE of 26.2% under P1 and 16.4% under P2 relative to CK (*p* < 0.05), with no significant difference between the two stress treatments. The average GPUE of CN 15 was significantly higher than that of SN 22, suggesting a greater capacity for internal phosphorus utilization under limited P supply.

## 3. Discussion

Developing crop genotypes that maintain productivity with reduced phosphorus (P) inputs is a critical goal for sustainable agriculture [36]. In the present study, we proposed a multi-stage screening pipeline that integrated percent tolerance to phosphorus deficiency (PTPD), principal component analysis, and a comprehensive evaluation index to identify reliable selection traits and phosphorus-efficient soybean genotypes.

The broad variation in PTPD and comprehensive evaluation (CE) values observed among 98 genotypes is consistent with extensive genetic diversity in soybean response to low-phosphorus stress, consistent with previous reports in various crops [33,37]. The majority of growth traits declined significantly under P deficiency, with photosynthetic rate being the most severely affected (average −32.4%) and displaying the highest diversity index. This observation reinforces the central role of photosynthesis in plant responses to P limitation [38], as P is essential for ATP synthesis, RuBP regeneration, and chloroplast integrity. A small number of positive PTPD values were observed, indicating better trait performance under low phosphorus than under control conditions. These cases may reflect compensatory growth triggered by mild stress [39,40] or the well-documented acceleration of reproductive development under phosphorus limitation [41,42]. Such positive values should not be taken to suggest that phosphorus deficiency is advantageous; rather, they point to genotype-specific adaptive responses that merit further investigation.

A key challenge in breeding for phosphorus efficiency is the identification of easily measurable, high-throughput traits that reliably reflect overall phosphorus efficiency. Through PCA, we reduced the dimensionality of ten growth parameters and identified five core traits—SPAD values at V3 and R1, photosynthetic rate at R1, shoot dry weight at R8, and seed number per plant at R8—that repeatedly emerged as dominant contributors in both Experiment I and Experiment II. The consistency of these results across two independent screenings strengthens the argument for their use in routine selection. SPAD values, in particular, are rapid and non-destructive measures of leaf chlorophyll content, which declines under P stress due to reduced protein synthesis and chloroplast development [43,44]. Similarly, shoot dry weight and seed number at maturity integrate the cumulative effects of P availability throughout the growth cycle and are agronomically relevant endpoints.

Although root architectural traits are often emphasized in P efficiency studies [45], their measurement is labor-intensive and poorly suited for large-scale screening. Moreover, Vandamme et al. [46] noted that increases in root-to-shoot ratio under low P often reflect stunted shoot growth rather than enhanced root proliferation, which further complicates the interpretation of root morphology alone as a selection criterion. Our results suggest that shoot-based physiological traits, particularly those linked to photosynthetic capacity and reproductive output, may serve as pragmatic selection criteria for identifying genotypes with superior internal phosphorus utilization efficiency. However, it should be noted that root traits were not directly assessed in this study, and the contribution of the root system to phosphorus efficiency remains unevaluated. The absence of root phenotyping is therefore acknowledged as a limitation, and future studies integrating both shoot and root phenotyping are warranted to validate these findings.

Among the six phosphorus-efficient genotypes identified in Experiment II, CN 15 was unique in achieving the highest comprehensive evaluation (CE) value in both experiments: 0.856 (rank 1/98) in Experiment I and 1.06 (rank 1/12) in Experiment II. This consistency across independent screening stages suggests that CN 15 integrates multiple tolerance-related traits more effectively than other genotypes tested. In Experiment III, CN 15 maintained significantly higher ET_0_/RC, φE_0_, and PIABS than SN 22 under all phosphorus levels (*p* < 0.05), and exhibited a 26.2% increase in GPUE under 0 µM KH_2_PO_4_ treatment. These results indicate that CN 15 sustains photosynthesis under low P through more efficient electron transport and light energy utilization [47,48] and superior internal P-utilization efficiency (PUtE). Although not every pairwise trait difference among the six efficient genotypes reached statistical significance, CN 15 uniquely combined the highest composite CE ranking across both experiments with the strongest physiological validation in Experiment III, supporting its selection as a promising candidate for further field evaluation.

It should be acknowledged that the reduced set of traits identified by PCA, though consistently contributing to the comprehensive evaluation, does not by itself establish their predictive value for independent genotype panels. The secondary screening employed two phosphorus levels (0 and 100 µM Pi), which better reflect gradual soil P depletion, and ranking genotypes by the sum of CE values across both stress levels helps avoid selecting lines that perform well only under extreme starvation. Furthermore, the three experiments were conducted in different years (2020–2023). While the screening pipeline was sequential and year-to-year environmental variation could not be statistically separated from the screening stage, the greenhouse conditions were managed consistently to minimize environmental fluctuations. The strong physiological contrasts observed between the two extreme genotypes (CN 15 and SN 22) provide encouraging biological support for the selected indicators; however, because only the extremes were validated, the resolution of these traits for discriminating intermediate phosphorus efficiency remains to be tested in a broader population.

In addition to the above considerations, sand culture differs from field soils in several key aspects that may affect phosphorus efficiency rankings. First, sand lacks the strong P-sorption capacity of many agricultural soils (e.g., Fe/Al oxides in acidic soils or Ca-bound P in calcareous soils), which can drastically reduce P availability and alter the relative importance of P uptake versus internal utilization traits. Second, the absence of a complex rhizosphere microbiome in sand eliminates potential plant–microbe interactions, such as mycorrhizal colonization or P-solubilizing bacteria, which are known to contribute to P acquisition under field conditions. Third, sand provides a homogeneous rooting environment with uniform P distribution, whereas field soils exhibit pronounced spatial heterogeneity in both P concentration and root accessibility. Finally, the controlled greenhouse environment does not capture fluctuations in temperature, moisture, and other abiotic stresses that can modulate plant responses to P limitation. Therefore, although sand-culture screening is effective for initial identification of physiological traits related to internal P utilization, the agronomic performance and ranking stability of the selected genotypes need to be validated under diverse field conditions before breeding programs can rely on these selections. Future multi-location and multi-year field evaluation is essential to confirm the stability of these genotypes’ phosphorus efficiency.

Beyond its immediate utility in phenotypic screening, the phenotyping pipeline—from PTPD standardization to CE calculation—may provide a foundation for integrating physiological screening with genomic tools. It integrates multiple tolerance-related traits into a single index. This makes it well-suited as a phenotypic target for genomic studies. In a typical genomic selection (GS) pipeline, the CE value can serve as the response variable, with genome-wide SNP markers (coded as 0, 1, 2) as predictors. Rather than building separate models for each of the ten traits—which may yield conflicting predictions—a single GS model based on CE can directly predict the overall low-P tolerance of breeding lines using marker data alone. Similarly, CE-based GWAS can detect genomic regions associated with comprehensive phosphorus efficiency, which may harbor regulatory genes that coordinate multiple downstream physiological responses rather than controlling a single trait. Although genomic data were not generated in the present study, the phenotyping framework established here—from PTPD standardization to CE calculation—provides a ready-to-use quantitative phenotype for future molecular dissection of phosphorus efficiency in soybean.

## 4. Materials and Methods

### 4.1. Plant Materials and Growth Conditions

All experiments were conducted at the Soybean Research Institute, Jilin Academy of Agricultural Sciences (Changchun, China) during the 2019–2023 growing seasons. Seeds of 98 major soybean genotypes cultivated in Jilin, Heilongjiang, and Inner Mongolia were obtained from the Institute’s resource-sharing platform. Before sowing, seeds were inoculated with Brady rhizobium japonicum strain LXB0002 (Leading Bio-agricultural Co., Ltd., Beijing, China) at a rate of 1.5 mL inoculant per 0.8 kg of seeds. Plants were grown in rectangular sand-filled boxes (312 × 117 × 29 cm) in a greenhouse under natural light supplemented with high-pressure sodium lamps to maintain a minimum photosynthetic photon flux density (PPFD) of approximately 500 µmol m^−2^ s^−1^ at canopy height, corresponding to a daily light integral (DLI) of 15–25 mol m^−2^ d^−1^ depending on seasonal solar radiation. The photoperiod was maintained at 14–16 h light/8–10 h dark, approximating the natural daylength during the growing season. Air temperature was maintained at 28 ± 2 °C (day)/20 ± 2 °C (night), and relative humidity averaged 60% (range: 45–75%). To minimize microenvironmental variation among boxes, the sand substrate was thoroughly homogenized before filling all boxes. All boxes were randomly repositioned within the greenhouse bay every two weeks to eliminate potential positional gradients. The sand substrate had the following properties: pH 7.16, available N 35.20 mg kg^−1^, total N 0.38 g kg^−1^, available P 0.59 mg kg^−1^, total P 0.08 g kg^−1^, available K 48.75 mg kg^−1^, total K 1.57 g kg^−1^.

A modified Hoagland nutrient solution was used, containing (in mM): 2.0 Ca(NO_3_)_2_, 0.75 K_2_SO_4_, 0.65 MgSO_4_, 0.08 Fe-EDTA, 10 × 10^−3^ H_3_BO_3_, 1 × 10^−3^ MnSO_4_, 0.5 × 10^−3^ CuSO_4_, 0.5 × 10^−3^ ZnSO_4_, and 0.05 × 10^−3^ (NH_4_)_6_Mo_7_O_24_. Phosphorus was supplied as KH_2_PO_4_ at three concentrations according to treatment. The pH was adjusted to 5.8 ± 0.1 daily. All reagents were obtained from Sinopharm Chemical Reagent Co., Ltd. (Shanghai, China). Boxes were watered every other day to maintain approximately 60% field capacity, monitored with a TDR 350 soil moisture meter (Spectrum Technologies, Inc., Aurora, IL, USA).

### 4.2. Experiment I: Preliminary Screening

From 2019 to 2021, ninety-eight genotypes were evaluated under two P treatments: CK (500 µM KH_2_PO_4_) and P1 (0 µM KH_2_PO_4_). Nutrient treatments were applied from the V1 stage onward. Fourteen genotypes were sown per box at a spacing of 21 cm between rows and 10 cm within rows. A panel of 98 soybean genotypes was evaluated for low-P tolerance over three consecutive growing seasons (2019, 2020, 2021), with each year serving as a complete block. Within each year, genotypes were grown under two P treatments (CK and P1) using a split-plot arrangement in a randomized complete block design with no within-year replication, with P treatment as the main plot and genotype as the subplot. Each P treatment main plot comprised seven sand-filled boxes, each containing 14 randomly assigned genotypes. Thus, all 98 genotypes were present in each year × P treatment combination. This design resulted in three independent biological replicates (n = 3 years) for each genotype × P treatment combination, where year served as the true replicate.

Ten growth parameters were measured: photosynthetic rate at R1 (X_1_), SPAD value at V3 (X_2_) and R1 (X_3_), shoot dry weight at R5 (X_4_), seed number per plant at R5 (X_5_), plant height at R5 (X_6_), shoot fresh weight at R5 (X_7_), plant height at R8 (X_8_), shoot dry weight at R8 (X_9_), and seed number per plant at R8 (X_10_). Growth stages were defined as follows: V3, third trifoliolate fully expanded; R1, beginning flowering (one open flower at any node on the main stem); R5, beginning seed (seed 3 mm long in a pod at one of the four uppermost nodes); and R8, full maturity (95% of pods with mature pod color). SPAD values were taken with a Minolta SPAD-502 m (Konica Minolta, Inc., Tokyo, Japan) on the youngest fully expanded trifoliate leaves between 09:00 and 11:00 on sunny days. Photosynthetic rate was measured on the same leaf type using an Li-6400 XT system (Li-Cor, Lincoln, NE, USA). The chamber conditions were controlled as follows: photosynthetic photon flux density (PPFD) was set to 1500 µmol m^−2^ s^−1^ using the built-in LED light source; reference CO_2_ concentration was maintained at 400 µmol mol^−1^; block temperature was set to 28 °C; and airflow rate was set to 500 µmol s^−1^. Relative humidity in the chamber ranged between 55 and 65%, corresponding to a leaf-to-air vapor pressure deficit (VPD) of approximately 1.5–2.0 kPa. At R5 and R8, plants were cut at the cotyledonary node to determine fresh and dry (oven-dried at 70 °C for 48 h) biomass and yield components.

### 4.3. Experiment II: Further Selection 

Experiment II was designed as a secondary evaluation to further discriminate phosphorus efficiency among the contrasting genotypes identified in Experiment I. It did not represent an independent validation panel but rather a refined screening step under multiple P levels. Based on CE values from Experiment I, the six most tolerant and six most sensitive genotypes were selected in 2022. They were subjected to three P treatments: CK (500 µM), P1 (0 µM), and P2 (100 µM KH_2_PO_4_), with three replicates in a split-plot design.

Genotypes were assigned to main plots and P treatments to subplots. Six genotypes per box were sown using the same spatial arrangement. Six key traits identified by PCA in Experiment I were recorded: photosynthetic rate at R1 (Y_1_), SPAD at V3 (Y_2_), SPAD at R1 (Y_3_), shoot dry weight at R5 (Y_4_), shoot dry weight at R8 (Y_5_), and seed number per plant at R8 (Y_6_).

Equal weights were assigned to CE values from the two P regimes. This is because, from a breeding perspective, target environments often involve variable rather than constant P stress, and genotypes that perform well under both severe and moderate limitation represent broader, more stable P efficiency. There was no a priori evidence that one stress level is biologically more informative than the other, making equal weighting the most neutral assumption. Differential weighting could be applied in future studies if a specific stress scenario is targeted.

### 4.4. Experiment III: Physiological Evaluation

In 2023, two contrasting genotypes—CN 15 (P-efficient) and SN 22 (P-inefficient)—were grown under the three P regimes in a split-plot design with three blocks. Genotypes were assigned to main plots and P treatments to subplots. At the R2 stage, OJIP chlorophyll fluorescence transients were measured on five fully dark-adapted leaves per treatment in each block using a PAM-2500 fluorometer (Walz, Effeltrich, Germany) after a 2 s saturating red light pulse (650 nm, 3000 µmol photons m^−2^ s^−1^). The five leaf-level measurements were averaged to provide a single value per block for statistical analysis. Thus, n = 3 in all physiological measurements represents three independent biological replicates (blocks). JIP-test parameters were calculated according to Strasser et al. [49], including TR_0_/RC (trapped energy flux per RC), ET_0_/RC (electron transport flux per RC), DI_0_/RC (dissipated energy flux per RC), φE_0_ (quantum yield of electron transport), VJ (relative variable fluorescence at J-step), and PIABS (performance index on absorption basis). At R8 maturity, grains were harvested, dried, weighed, and ground. Phosphorus concentration was determined by ICP-OES (PerkinElmer Optima 8000, PerkinElmer, Inc., Waltham, MA, USA) after microwave digestion (6 mL HNO_3_ + 2 mL H_2_O_2_). Grain phosphorus utilization efficiency (GPUE) was calculated as grain dry weight (g plant^−1^) divided by grain P content (mg plant^−1^). Both dry weight and P content were determined from the same blocks, maintaining a consistent replication structure with n = 3 biological replicates.

### 4.5. Data Analysis

#### 4.5.1. PTPD Calculation

The percent tolerance to phosphorus deficiency (PTPD) was calculated for each trait as:

(1) $$\mathrm{PTPD}\;=\frac{X_{P}-X_{CK}}{X_{CK}}\times 100\%$$

where *X*_P_ is the trait value under phosphorus-deficient conditions (P1 or P2 treatment), and *X*_CK_ is the trait value under control conditions (500 µM KH_2_PO_4_).

#### 4.5.2. Principal Component Analysis and Standardization

Before PCA, PTPD values were standardized to zero mean and unit variance using Z-score transformation: $Z=\left(X-\bar{X}\right)/\sigma$, where $\bar{X}$ is the trait mean and σ is the standard deviation across the 98 genotypes. PCA was then performed on the correlation matrix of the ten standardized PTPD variables. Principal components (PCs) with eigenvalues > 1 were retained. The suitability of the data for PCA was assessed using the Kaiser–Meyer–Olkin (KMO) measure and Bartlett’s test of sphericity.

#### 4.5.3. Comprehensive Evaluation (CE) and Membership Function

Comprehensive evaluation (CE) values were derived using a weighted membership function approach. For each retained PC, the membership function value (*M_ij_*) of each genotype was calculated using the fuzzy membership formula:

(2) $${\;M}_{ij}=\;\frac{F_{ij}-F_{min,j}}{F_{max,j}-F_{min,j}}$$

where *F_ij_* is the factor score of the i-th genotype on the j-th PC, and $F_{\max,j}$ and $F_{\min,j}$ are the maximum and minimum PC scores across all genotypes for that component. The weight ($W_{j}$) of each PC was defined as the proportion of variance explained:

Weights (*W*_*j*_) for each PC were defined as the proportion of the total variance explained by that component:

(3) $${\;W}_{j}=\;\frac{\lambda_{j}}{\sum \lambda }$$

where $\lambda_{j}$ is the eigenvalue of the j-th PC and ∑λ is the sum of eigenvalues of all retained PCs. The comprehensive evaluation (CE*_i_*) value for the i-th genotype was then calculated as the weighted sum of membership values:

(4) $${CE}_{i}=\;\sum_{j=1}^{m}W_{j}\times M_{ij}$$

where *m* is the number of retained PCs. A higher CE value indicates stronger overall tolerance to phosphorus deficiency.

#### 4.5.4. Shannon–Wiener Diversity Index (H′)

To quantify the phenotypic variation in each growth parameter in response to phosphorus deficiency across genotypes, the Shannon–Wiener diversity index (H′) was calculated based on the PTPD values. For each parameter, the range of PTPD across the 98 genotypes was divided into ten equal-interval classes. The relative frequency (*p_k_*) of genotypes falling into the *k*th class was then determined. The diversity index was computed as:

(5) $$H^{\prime }=-\sum_{k=1}^{10}p_{k}ln(p_{k})$$

where $p_{k}=n_{k}/N$, with $n_{k}$ being the number of genotypes in the *k*th class and N=98 the total number of genotypes. If $p_{k}=0$, the term $p_{k}ln(p_{k})$ was set to zero. A higher H′ value indicates a more dispersed distribution of PTPD values, reflecting greater genotypic diversity for the trait under phosphorus deficiency stress. This index provides a standardized, non-parametric measure of trait variability that complements conventional parametric statistics.

#### 4.5.5. Cluster Analysis

Hierarchical cluster analysis was performed on the CE values using Ward’s method with squared Euclidean distance. Ward’s method was chosen because it minimizes the increase in within-cluster sum of squares at each fusion step, producing compact, well-separated clusters suitable for genotype classification. The cophenetic correlation coefficient was calculated to assess the fidelity of the dendrogram to the original distance matrix. The optimal number of clusters was determined by examining the fusion coefficient schedule, the pseudo-F statistic (PSF), and the R-squared (RSQ) values. The complete CE ranking, cluster membership, and tolerance category for all 98 genotypes are provided in Supplementary Table S1.

#### 4.5.6. Mixed Model Analysis of Split-Plot Experiments

Data from the split-plot experiments were analyzed using the linear mixed model procedure (MIXED) in SPSS 22.0. In Experiment I, a split-plot design was used, with phosphorus treatment (P) as the whole-plot factor and genotype (G) as the subplot factor. Three blocks were established, with each block corresponding to one complete growing season (three consecutive years). The linear mixed model included P, G, and their interaction (P × G) as fixed effects. The block and the block × P interaction were included as random effects, with block × P serving as the whole-plot error term for testing the main effect of P. Regarding the potential box-to-box variation within each block × P combination in Experiment I, the seven boxes were considered physical subdivisions of the same main plot. Given that (i) the sand substrate was thoroughly homogenized before filling, (ii) boxes were randomly repositioned every two weeks to minimize positional gradients, and (iii) genotypes were completely randomized across boxes, any systematic box-to-box variation was expected to be minimal and, if present, would be absorbed by the Block × P random intercept. Therefore, Box was not included as an additional random effect; its minimal variation was pooled into the residual error term to avoid over-parameterization. The same rationale applied to Experiments II and III, where boxes were also treated as technical replicates. In both Experiment II and Experiment III, genotype (G) was the whole-plot factor and phosphorus treatment (P) was the subplot factor, with three blocks (replicates). The model included G, P, and their interaction (G × P) as fixed effects. Block and the block × G interaction were specified as random effects, with block × G serving as the whole-plot error term for testing the main effect of G. The residual variance provided the subplot error term for testing P and the G × P interaction. Denominator degrees of freedom were estimated using the Satterthwaite approximation. Mean comparisons were conducted via the EMMEANS subcommand with Bonferroni correction (or Tukey’s HSD). All tests were performed at α = 0.05.

In addition to the primary mixed-model analysis for testing treatment and interaction effects, a separate one-way ANOVA (genotype as a fixed factor) was performed on the PTPD values for each of the ten growth parameters to assess genotypic variation within individual traits. The resulting F and *p* values and genotype degrees of freedom (df = 97) are reported in Table 1. Fisher‘s LSD test was then applied to conduct pairwise genotype comparisons within each parameter, and the corresponding LSD bars were used in Figure 1 solely for visualizing the degree of genotypic dispersion. These analyses are independent of the primary split-plot mixed model.

Statistical analyses were conducted using SPSS 22.0 (IBM Corp., Armonk, NY, USA)., DPS 22.05 (Data Processing System, Hangzhou Ruifeng Information Technology Co., Ltd., Hangzhou, China), and OriginPro 2021 (OriginLab Corp., Northampton, MA, USA).

## 5. Conclusions

In conclusion, our multi-stage screening approach based on percent tolerance to phosphorus deficiency (PTPD) successfully identified photosynthetic rate, SPAD chlorophyll index at early and mid-reproductive stages, shoot dry weight at maturity, and seed number per plant as informative indicators for assessing soybean low-P tolerance under controlled conditions. The genotype CN 15 consistently exhibited superior phosphorus utilization efficiency and PSII functionality in sand culture, suggesting its potential as a candidate for further evaluation. However, as all experiments were conducted in a greenhouse using sand culture, the agronomic relevance of these traits and the selected genotypes remain to be validated under multi-location and multi-year field trials.

## Figures and Tables

**Table 1:** The effect of phosphorus deficiency on 10 growth parameters of 98 soybean (*Glycine max* L.) genotypes in Experiment I expressed as PTPD (%).

PTPD	X_1_	X_2_	X_3_	X_4_	X_5_	X_6_	X_7_	X_8_	X_9_	X_10_
Mean	−32.4	−3.9	−1.4	−11.1	−10.8	−10.9	−12.9	−15.1	−15.5	−14.9
Min	−77.9	−43.6	−13.1	−75.6	−78.9	−45.0	−80.5	−78.5	−80.1	−100.0
Max	4.5	4.2	2.4	17.8	19.4	6.4	19.8	5.0	30.3	31.0
CV(%)	66.2	197.0	255.3	169.5	176.8	113.0	164.4	105.0	144.8	155.1
*F* value	3.476	4.08	1.417	5.446	9.687	3.514	48.433	11.499	230.79	8.851
*p* value	0.0001	0.0001	0.0209	0.0001	0.0001	0.0001	0.0001	0.0001	0.0001	0.0001

The parameters are: X_1_, photosynthetic rate at R1 stage; X_2_, SPAD value at V3 stage; X_3_, SPAD value at R1 stage; X_4_, shoot DW at R5 stage; X_5_, seed number per plant at R5 stage; X_6_, plant height at R5 stage; X_7_, shoot FW at R5 stage; X_8_, plant height at R8 stage; X_9_, shoot DW at R8 stage; X_10_, seed number per plant at R8 stage. For traits where PTPD values included negative numbers and the mean was negative, the absolute value of CV is reported to indicate relative variability. The *F* and *p* values were obtained from one-way ANOVA of PTPD values for each individual trait, with genotype as the fixed factor (df = 97).

**Table 2:** The corresponding feature vectors of 5 principal components on 10 growth characters, and their contribution rate (R) and weight coefficient (W) in Experiment I.

Principal Component	X_1_	X_2_	X_3_	X_4_	X_5_	X_6_	X_7_	X_8_	X_9_	X_10_	R	W
C_1_	0.144	−0.035	−0.018	0.473	0.441	0.366	0.455	0.268	0.283	0.259	38.124	0.434
C_2_	0.071	−0.026	0.133	−0.201	−0.264	−0.236	−0.248	0.410	0.545	0.533	19.625	0.223
C_3_	0.583	−0.421	0.646	0.005	0.097	−0.068	−0.052	−0.209	−0.063	−0.038	12.018	0.137
C_4_	0.250	0.865	0.238	0.108	0.084	−0.217	0.038	−0.239	0.061	0.075	9.858	0.112
C_5_	−0.663	0.080	0.698	0.022	0.010	0.234	−0.008	0.081	0.032	−0.063	8.247	0.094

The parameters are: X_1_, photosynthetic rate at R1 stage; X_2_, SPAD value at V3 stage; X_3_, SPAD value at R1 stage; X_4_, shoot DW at R5 stage; X_5_, seed number per plant at R5 stage; X_6_, plant height at R5 stage; X_7_, shoot FW at R5 stage; X_8_, plant height at R8 stage; X_9_, shoot DW at R8 stage; X_10_, seed number per plant at R8 stage.

**Table 3:** The corresponding feature vectors of four principal components on six growth characters, and their contribution rate (R) and weight coefficient (W) in Experiment II.

Principal Component	Y_1_	Y_2_	Y_3_	Y_4_	Y_5_	Y_6_	R	W
CC_1_	0.310	0.078	0.220	−0.338	0.620	0.592	31.988	0.371
CC_2_	0.600	−0.144	0.470	0.514	0.151	−0.334	22.879	0.265
CC_3_	0.088	0.845	0.350	−0.258	−0.217	−0.207	17.696	0.205
CC_4_	0.258	0.459	−0.701	0.402	0.265	0.018	13.649	0.158

The parameters are: Y_1_, photosynthetic rate at R1 stage; Y_2_, SPAD values at V3 stage; Y_3_, SPAD values at R1 stage; Y_4_, shoot DW at R5 stage; Y_5_, shoot DW at R8 stage; Y_6_, seed number per plant at R8 stage.

**Table 4:** Factor scores (F_ij_), membership function values (M_ij_), and comprehensive evaluation (CE) values for P1 and P2 treatments in Experiment II.

Genotypes	F_1_		F_2_		F_3_		F_4_		M_1_		M_2_		M_3_		M_4_		CE	
	P1	P2	P1	P2	P1	P2	P1	P2	P1	P2	P1	P2	P1	P2	P1	P2	P1	P2
CN 15	1.52	0.32	0.05	−0.04	0.56	0.14	0.94	1.13	0.50	0.32	0.50	0.49	0.63	0.53	0.75	0.80	0.57	0.49
JN 36	−1.15	4.97	−0.35	0.07	1.15	−1.90	0.07	0.48	0.11	1.00	0.43	0.51	0.76	0.08	0.51	0.63	0.39	0.62
JIN 23	0.81	−1.64	2.73	0.59	1.16	−0.15	−1.04	0.46	0.39	0.04	1.00	0.60	0.76	0.47	0.21	0.62	0.60	0.37
JY 69	0.05	0.23	−0.57	1.04	0.63	0.65	1.12	0.47	0.28	0.31	0.39	0.69	0.64	0.65	0.80	0.62	0.47	0.53
KX 6	0.26	0.12	−1.02	−0.30	2.24	1.47	−0.06	−0.31	0.31	0.29	0.30	0.44	1.00	0.83	0.48	0.41	0.48	0.46
JY 95	0.22	0.83	−0.27	0.74	1.15	0.46	0.04	−0.69	0.31	0.40	0.44	0.63	0.76	0.60	0.51	0.31	0.47	0.49
HF 25	−1.21	1.21	−1.26	−0.43	−0.35	0.28	1.59	−0.99	0.10	0.45	0.26	0.41	0.42	0.56	0.93	0.22	0.34	0.43
JIN 18	0.19	0.16	−0.95	−0.25	0.03	−0.59	−0.03	−0.56	0.30	0.30	0.32	0.45	0.51	0.37	0.49	0.34	0.38	0.36
OK 25	−0.15	0.26	1.19	−1.13	−0.09	−0.56	−0.55	−0.18	0.26	0.31	0.72	0.28	0.48	0.38	0.34	0.44	0.44	0.34
JN 27	−1.46	−1.86	1.01	2.70	−0.78	−1.09	−0.24	1.85	0.06	0.01	0.68	0.99	0.33	0.26	0.43	1.00	0.34	0.48
JIN 21	−0.63	−0.45	0.12	−0.01	−2.26	−0.70	−0.88	−1.37	0.18	0.21	0.52	0.49	0.00	0.35	0.26	0.12	0.25	0.30
SN 22	−0.70	−1.90	−2.66	−0.99	−0.88	−0.55	0.57	−1.81	0.17	0.00	0.00	0.31	0.31	0.38	0.65	0.00	0.23	0.16

CK, 500 µM KH_2_PO_4_; P1, 0 µM KH_2_PO_4_; P2, 100 µM KH_2_PO_4_.

## Data Availability

The original contributions presented in the study are included in the article and its Supplementary Materials. Further inquiries can be directed to the corresponding authors.

## Supplementary Materials

The following supporting information can be downloaded at: https://www.mdpi.com/article/10.3390/plants15152408/s1. Table S1, Comprehensive evaluation parameters and tolerance classification of 98 soybean genotypes under phosphorus deficiency stress (P1) in Experiment I. Table S2. Hierarchical cluster agglomeration schedule for 98 soybean genotypes based on comprehensive evaluation (CE) values in Experiment I. Table S3. Hierarchical cluster agglomeration schedule for 12 soybean genotypes based on comprehensive evaluation (CE) values in Experiment II.

## Abbreviations

The following abbreviations are used in this manuscript:

GPUE	Grain phosphorus utilization efficiency
PTPD	Percent tolerance to phosphorus deficiency
PUpE	Phosphorus uptake efficiency
PUtE	Phosphorus utilization efficiency
